# P-836. Routine Admission Urinalysis Has Low Clinical Utility in Psychiatric Hospitalizations

**DOI:** 10.1093/ofid/ofaf695.1044

**Published:** 2026-01-11

**Authors:** Tri Pham, Alice Bewley, Sena Sayood

**Affiliations:** Washington University in St. Louis School of Medicine, St. Louis, MO; Washington University School of Medicine in St. Louis, St. Louis, Missouri; Washington University School of Medicine, St. Louis, Missouri

## Abstract

**Background:**

Guidelines recommend against screening for and treating asymptomatic bacteriuria (ASB), a driver of inappropriate antibiotic use (IAU). Despite this, urinalyses (UAs) are often obtained at time of psychiatric admissions even in asymptomatic patients, potentially leading to unnecessary treatment. We assessed the utility of admission UAs in psychiatric hospitalizations and identified factors leading to IAU.

**Methods:**

We extracted psychiatric hospitalization records from January 2020 through December 2024 at a large academic hospital system. We defined admission UAs as those collected within 48-hours before and after arrival to the psychiatric unit to account for transfer or specimen collection delays. A positive UA was defined as the presence of pyuria or a culture (UC) with > 100,000 colony-forming units. We defined a urinary tract infection (UTI) as a positive UA with localizing symptoms and excluded a UTI in those unable to report symptoms if they had no systemic signs of infection. We considered antibiotic use appropriate if a patient met the criteria for UTI or had a positive UA during pregnancy. Uni- and multivariable logistic regression models estimated using generalized estimating equations were fitted to IAU as the dependent variable, using sandwich-type standard errors to account for potential correlation from multiple encounters per individual.

**Results:**

Among 8,891 patients with 13,495 psychiatric hospitalizations, 10,824 (80.2%) received admission UAs, with 1,382 (12.7%) having positive UAs. Of these, 635 (45.9%) were treated with antibiotics, but 463 (72.9%) of those treatments were inappropriate. Only 172 admission UAs (1.6%) led to treatment of a true UTI or ASB in pregnancy. Older age, female gender, leukocytosis, and diagnoses of schizophrenia, substance use, and bipolar disorders emerged as significant factors associated with IAU. In a subgroup analysis limited to encounters with reflex-to-culture testing, a positive UC was also a significant predictor of IAU.

**Conclusion:**

Routine admission UAs for psychiatric hospitalizations likely contribute to IAU. We identified factors associated with IAU that can potentially inform stewardship efforts aimed at encouraging judicious use of UAs and reservation of testing for those with high clinical suspicion for a UTI.

**Disclosures:**

All Authors: No reported disclosures

